# Cell death–associated lipid droplet protein CIDE-A is a noncanonical marker of endoplasmic reticulum stress

**DOI:** 10.1172/jci.insight.143980

**Published:** 2021-04-08

**Authors:** Yoshiaki Morishita, Aaron P. Kellogg, Dennis Larkin, Wei Chen, Suryakiran Vadrevu, Leslie Satin, Ming Liu, Peter Arvan

**Affiliations:** 1 Division of Metabolism, Endocrinology & Diabetes, Department of Internal Medicine, University of Michigan, Ann Arbor, Michigan, USA.; 2 Division of Diabetes, Department of Internal Medicine, Aichi Medical University, Aichi, Japan.; 3 Department of Pharmacology, University of Michigan Medical Center, Ann Arbor, Michigan, USA.; 4 Department of Endocrinology & Diabetes, Tianjin Medical University, Tianjin, China.

**Keywords:** Endocrinology, Cell stress, Protein misfolding, Protein traffic

## Abstract

Secretory protein misfolding has been linked to ER stress and cell death. We expressed a *TG^rdw^* transgene encoding TG-G(2298)R, a misfolded mutant thyroglobulin reported to be linked to thyroid cell death. When the *TG^rdw^* transgene was expressed at low level in thyrocytes of *TG^cog/cog^* mice that experienced severe ER stress, we observed increased thyrocyte cell death and increased expression of CIDE-A (cell death-inducing DFFA-like effector-A, a protein of lipid droplets) in whole thyroid gland. Here we demonstrate that acute ER stress in cultured PCCL3 thyrocytes increases *Cidea* mRNA levels, maintained at least in part by increased mRNA stability, while being negatively regulated by activating transcription factor 6 — with similar observations that ER stress increases *Cidea* mRNA levels in other cell types. CIDE-A protein is sensitive to proteasomal degradation yet is stabilized by ER stress, and elevated expression levels accompany increased cell death. Unlike acute ER stress, PCCL3 cells adapted and surviving chronic ER stress maintained a disproportionately lower relative mRNA level of *Cidea* compared with that of other, classical ER stress markers, as well as a blunted *Cidea* mRNA response to a new, unrelated acute ER stress challenge. We suggest that CIDE-A is a novel marker linked to a noncanonical ER stress response program, with implications for cell death and survival.

## Graphical abstract

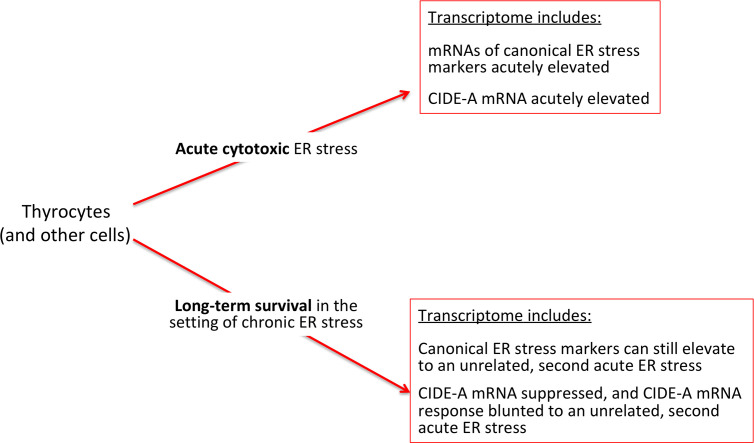

## Introduction

The thyroglobulin (*TG*) gene is the most highly expressed gene of the thyroid gland, and TG protein makes up more than 50% of all thyroidal protein. In order to serve as substrate for thyroid hormone synthesis, the TG protein must be exported through the secretory pathway (1). Misfolding of mutant TG in the ER is an established cause of congenital hypothyroidism (2), which, via endocrine feedback, triggers an increase in circulating levels of thyroid-stimulating hormone (TSH) released from the anterior pituitary. In this disease (in both humans and animal models), thyroid follicular (thyrocyte) cell growth (3) and thyrocyte cell death (4) take place simultaneously in different cells of the same thyroid gland. This behavior suggests that affected cells in the same tissue exhibit a range of (successful and unsuccessful) attempts in adaptive response to chronic ER stress. Such a finding is consistent with the broader notion that a myriad of prosurvival and prodeath signals are generated as downstream consequences of ER stress (5), and there are likely to be both complex (and possibly subtle) changes in the profile and balance of these activities that trigger death in some cells while allowing survival and even proliferation of adjacent cells of the same cell type.

In the case of the thyroid gland, the net balance of thyroid cell growth and death determines the potential for development of a goiter (which signifies net growth). Thus, in congenital hypothyroidism caused by mutant *TG*, the ultimate size of the goiter serves as a physiologically integrated readout of the balance of many hundreds of thousands of individual cell fate decisions. One of the striking outcomes of this balance is observed when comparing the congenital goiter (*cog/cog*) mouse and the dwarf (*rdw/rdw*) rat, both of which are caused by a single homozygous missense mutation of *TG*. The mutation in each case resides in the same ChEL (CholinEsterase-Like) domain, just 35 amino acids apart, within the 2765 residue TG protein (Supplemental Figure 1A; supplemental material available online with this article; https://doi.org/10.1172/jci.insight.143980DS1). However, the *cog/cog* mouse generates an enlarged thyroid (goiter, ref. 6), whereas the *rdw/rdw* rat develops atrophy of the thyroid gland (7).

In an attempt to understand this difference, our initial animal studies have led us to investigate CIDE-A (cell death-inducing DFFA-like effector-A) (8), which is an established protein of lipid droplets (9, 10). Herein, we describe the regulation of *Cidea* mRNA and protein in PCCL3 (cell line of cultured rat) thyrocytes challenged with acute and chronic ER stress. Specifically, we report that CIDE-A is a gene product linked to a noncanonical acute ER stress response, leading us to consider that CIDE-A may contribute to the complex cell fate program of ER-stressed thyrocytes as well as other cell types.

## Results

### Identification of Cidea in the thyroid gland of animals experiencing toxic ER stress.

**Figure 1:**
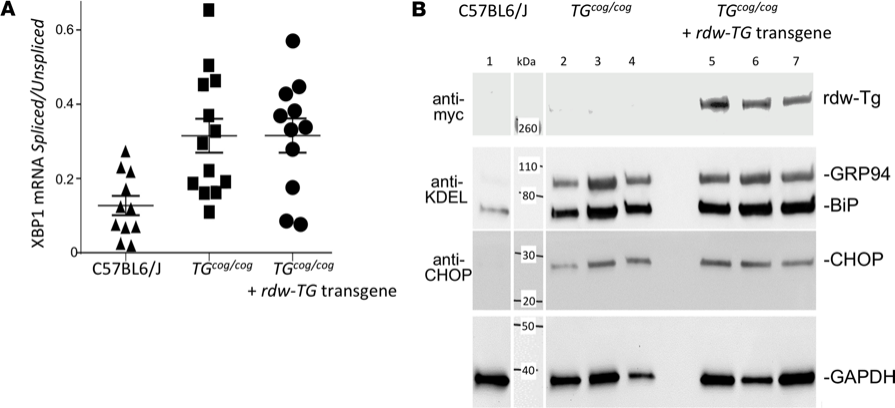
ER stress in *TG^cog/cog^* mice lacking or bearing the *TG^rdw^* transgene. (**A**) Thyroid glands were dissected and RNA prepared and reverse-transcribed from hypothyroid 2-month-old C57BL/6J or *TG^cog/cog^* mice ± the *TG^rdw^* transgene driven by the *TG* promoter (*n* ≥ 11 animals per group). Spliced/unspliced *Xbp1* mRNA quantitative reverse-transcribed PCR (qRT-PCR) is shown; each point is a different animal (both males and females were used). *TG^cog/cog^* mice lacking (0.32 ± 0.05) or bearing the *TG^rdw^* transgene (0.32 ± 0.05) both had significantly higher *Xbp1* splicing than was detected in control thyroid glands (0.12 ± 0.02). *P* < 0.05. (**B**) *Upper:* Anti-myc immunoblot of rdw-TG in thyroid glands of the genotypes above (2-month-old *TG^cog/cog^* mice ± transgene); each lane is a different animal. *Middle:* Anti-KDEL and anti-CHOP immunoblotting from the same samples. *Lower:* GAPDH is a loading control. The lane at far left is thyroid tissue from C57BL/6J run on the same gel and transfer membrane; adjacent molecular weight markers are shown. This experiment was repeated 3 times.

**Figure 2:**
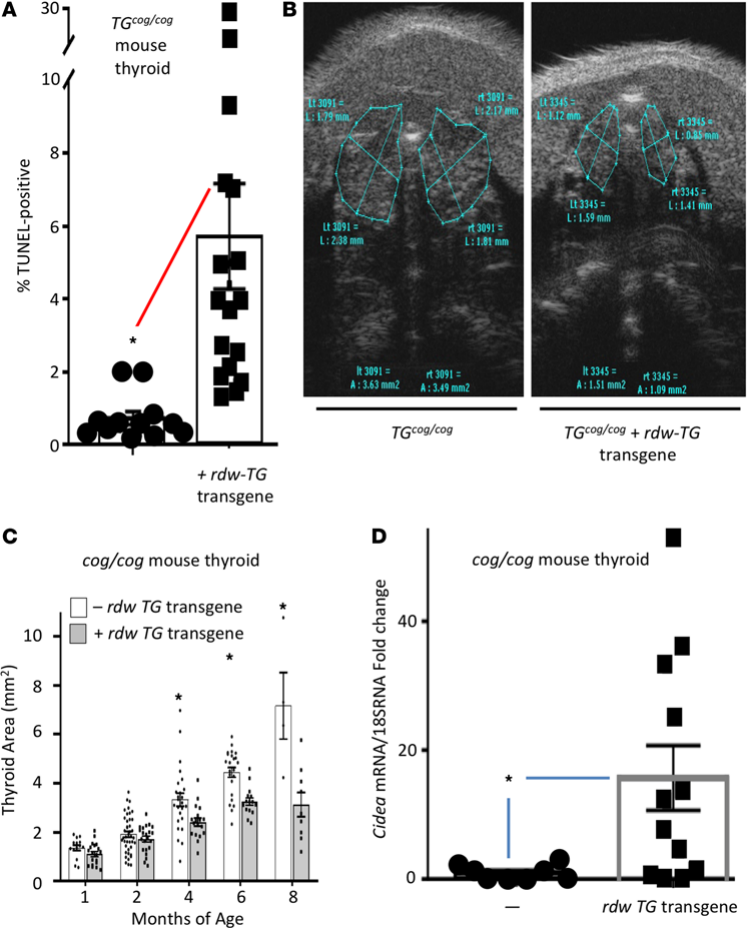
The *TG^rdw^* transgene limits net thyroid growth in *TG^cog/cog^* mice; the nontransgenic animals have less cell death and less thyroidal expression of *Cidea* mRNA. (**A**) TUNEL staining (% of DAPI-positive nuclei) in thyroid gland sections of 6-month-old *TG^cog/cog^* mice lacking (0.5 ± 0.1%, *n* = 12) or bearing the *TG^rdw^* transgene (5.9 ± 1.8%; each point represents a different animal). (**B**) Ultrasound images demonstrating thyroid area (6-month-old hypothyroid littermate animals of the genotypes shown). (**C**) Goiter growth quantified longitudinally by neck ultrasound in a cohort of 4 or more *TG^cog/cog^* and 9 or more transgenic *TG^cog/cog^* + *TG^rdw^* mice for up to 8 months. Error bars represent mean ± SEM; *P < 0.05 (2-tailed *t* test) in *TG^cog/cog^* mice versus their transgenic counterparts, beginning at 4 months of age. (**D**) qRT-PCR analysis of *Cidea* mRNA levels in hypothyroid 4-month-old *TG^cog/cog^* lacking or bearing the *TG^rdw^* transgene, with data expressed as fold change. Error bars represent mean ± SEM, *n* = 12 animals; *P < 0.05 between the 2 groups (Mann-Whitney *U* test).

Some patients suffering from congenital hypothyroidism with defective TG may develop thyroid gland overgrowth (goiter, ref. 11) whereas others may not. The homozygous *TG^cog/cog^* mouse encodes expression of misfolded TG that is defective for export from the ER, triggering thyrocyte ER stress throughout life (12, 13) even as the thyroid gland continues to grow. In contrast, the *TG^rdw/rdw^* rat expresses homozygous misfolded mutant TG that is also defective for ER export (14, 15) accompanied by chronic thyrocyte ER stress (16, 17), yet it develops an atrophic thyroid gland (7) with associated thyroid cell death (4). Given that the 2 missense substitutions are so proximate within the TG-ChEL domain, the disparate phenotypes are puzzling. To begin to study the problem, we developed a *TG^rdw^* transgene (Supplemental Figure 1, A and B) in which the *TG* promoter drives thyroid-specific expression (18) of the mutant TG protein (bearing a triple-Myc-epitope tag). Transgenic animals were identified by tail vein PCR (Supplemental Figure 1C); the transgene was expressed at a low level (≤1% of endogenous *TG* mRNA in *TG^cog/cog^* mice) such that total *TG* mRNA in the thyroid of transgenic animals was unchanged from that in *TG^cog/cog^* mice (Supplemental Figure 2A), and both sets of 4-month-old animals had primary hypothyroidism (with circulating T_4_ < half of control animals and circulating TSH elevated more than an order of magnitude and no significant differences between them). Nevertheless, thyroidal rdw-TG protein expression was confirmed in transgenic animals (Supplemental Figure 2B). Without the transgene, *TG^cog/cog^* animals already exhibited massive ER distension as reported previously (19) — this was also found in the transgenic *TG^cog/cog^* mice, as demonstrated by immunofluorescence of the ER marker immunoglobulin heavy chain binding protein (BiP) (Supplemental Figure 2C). Without the transgene, *TG^cog/cog^* animals exhibited dramatic ER stress as reported previously (13) — comparable ER stress was also found in transgenic *TG^cog/cog^* mice as demonstrated by X-box binding protein-1 (XBP1) splicing (Figure 1A) with upregulation of ER chaperones, as well as CCAAT-enhancer-binding protein homologous protein (CHOP) (Figure 1B). Both sets of *TG^cog/cog^* animals exhibited positive TUNEL staining in the thyroid gland, but TUNEL-positive thyroid cell death in the animals bearing the *rdw-TG* transgene was detectably greater (Figure 2A), and, as measured by live animal thyroid ultrasound (Figure 2B), expression of the transgene limited the magnitude of thyroid gland growth (Figure 2C).

In an early analysis, we prepared RNA from *TG^cog/cog^* ± *TG^rdw^* transgene (*n* = 4 per group) and performed a simple quantitative PCR (qPCR) microarray of cell death–related mRNAs, accompanied by positive and negative controls. Of the 86 gene products analyzed, the most upregulated mRNA found in the thyroids of *TG^cog/cog^* + *rdw-TG* was *Cidea* (Supplemental Figure 3A). Although there was considerable variability between individual animals, by qRT-PCR we independently confirmed an average 15-fold elevation of *Cidea* mRNA in the thyroid glands of transgenic animals (Figure 2D).

### Examination of Cidea mRNA response to ER stress in cell culture.

**Figure 3:**
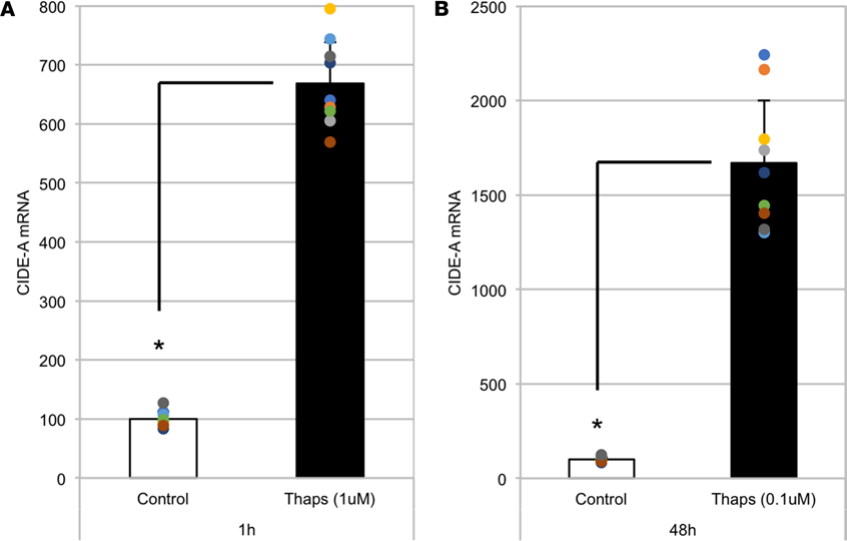
In the PCCL3 rat thyrocyte cell line, *Cidea* is induced by acute ER stress. *Cidea* mRNA levels (normalized to hypoxanthine phosphoribosyltransferase 1 [*Hprt1*]) in PCCL3 cells at 1 hour after treatment with high-dose thapsigargin (Thaps, 1 μM, **A**) or 48 hours after treatment with low-dose Thaps (0.1 μM, **B**). The data show the mean (from 3 independent experiments in which each sample had 3 biological replicates) ± SD; *P < 0.05 versus vehicle (DMSO) control (1-way ANOVA).

**Figure 4:**
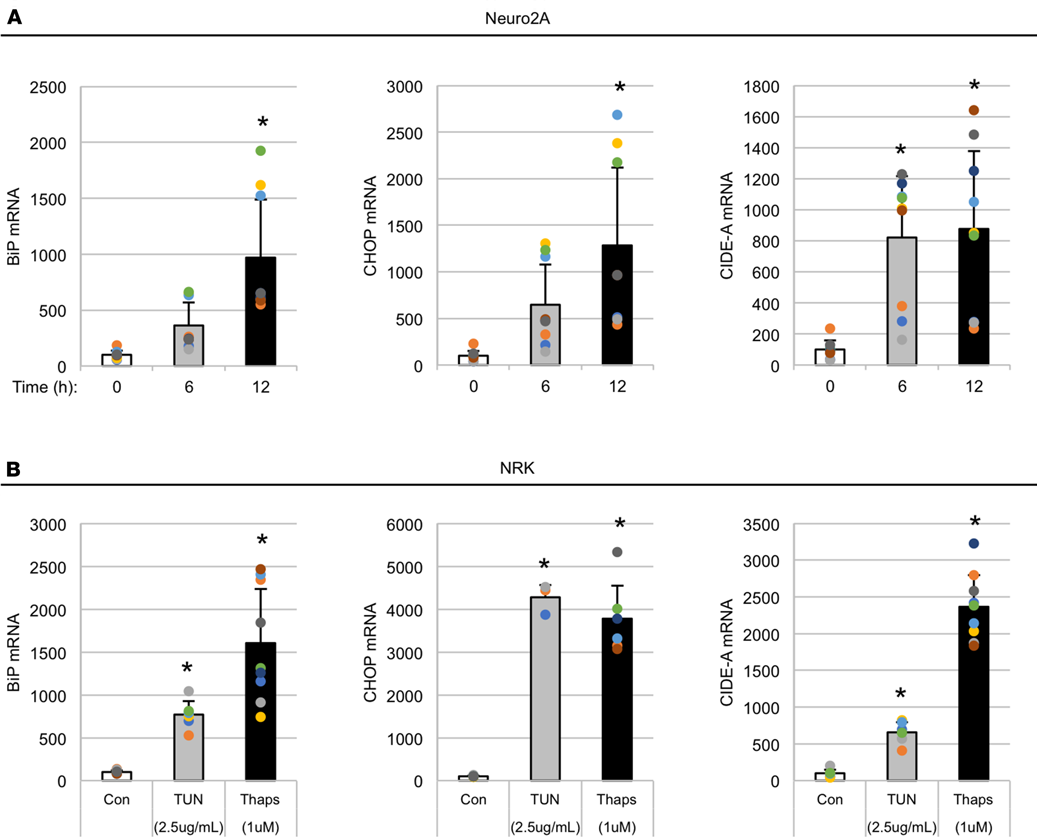
*Cidea* is induced by acute ER stress in nonadipocyte, nonthyroid cell lines. (**A**) Neuro2A cells were treated with 0.1 μM Thaps either 12 hours, or 6 hours, before lysis; untreated cells served as 0-hour control. At time 0, all cells were lysed and mRNA levels measured by qRT-PCR (normalized to 18S rRNA). The data represent the mean (from 3 biological replicates) in each of 3 independent experiments, ± SD (1-way ANOVA). (**B**) NRK cells were plated 24 hours before beginning the experiment. ER stress agents were added at time 0, and the cells were lysed at 48 hours and mRNA levels measured by qRT-PCR (normalized to *Hprt1*). The data represent the mean ± SD(from 3 independent biological replicates) with experiments done twice (TUN) or thrice (Thaps). * *P* < 0.05 versus 0 hours or control (Con, DMSO) (1-way ANOVA).

Normally, *Cidea* gene expression is enriched in adipocytes and other cell types that contain abundant lipid droplets (9), so it initially seemed surprising to detect an increase in *Cidea* in the ER-stressed thyroid gland. Unfortunately, we could find no reliable antibody to examine endogenous CIDE-A protein expression in mouse tissues. Thus, to investigate the effects of ER stress on *Cidea* expression specifically in thyrocytes, we began by challenging the PCCL3 (rat thyrocyte) cell line with either high-dose thapsigargin (Thaps, 1 μM) for 1 hour or low-dose Thaps (0.1 μM) for 2 days. Both ER stress challenges resulted in a dramatic increase in *Cidea* mRNA levels (Figure 3, A and B). Similar responses in PCCL3 cells were seen even after 6 hours of TSH withdrawal (data not shown); moreover, Thaps treatment induced the expression of *BiP*, *Chop*, and *Cidea* in Neuro2A cells (a neuronal cell line, Figure 4A), and similar results with Thaps (and another ER stress agent, tunicamycin; TUN) were also observed in NRK cells (a kidney cell line, Figure 4B). Thus, the effect of acute ER stress to stimulate *Cidea* mRNA levels is not a feature that requires ongoing TSH stimulation of thyrocytes, nor is it even unique to thyrocytes.

**Figure 5:**
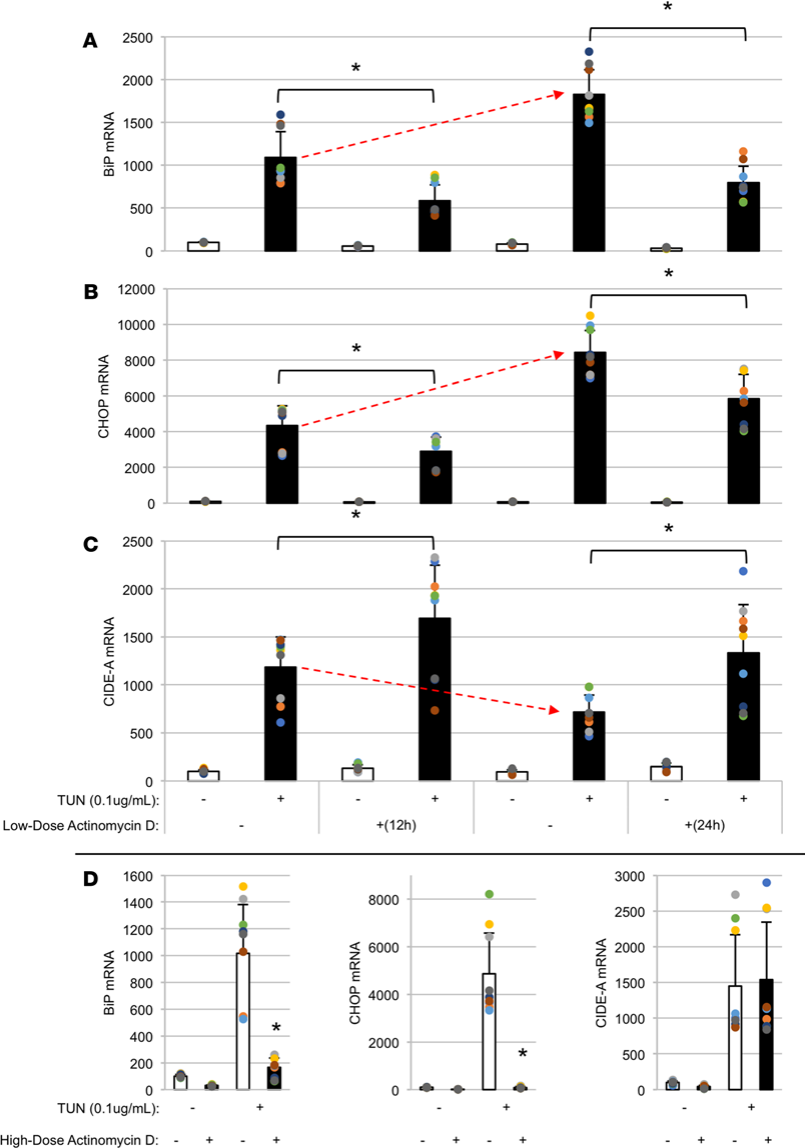
In response to acute ER stress, *Cidea* mRNA elevation is maintained, at least, in part, by enhanced mRNA stability. Replicate wells of PCCL3 cells were either untreated or treated with tunicamycin (TUN 0.1 μg/mL) at time = –12 hours or –24 hours. One set of wells were cotreated with a low dose of the transcription blocker, actinomycin D (5 ng/mL), as indicated. At time 0, the cells were lysed, and mRNA levels were measured by qRT-PCR for *BiP* (**A**), *Chop* (**B**), or *Cidea* (**C**). The data show the mean ± SD (from 3 independent experiments in which each sample had 3 biological replicates); *P < 0.05 TUN + actinomycin D treatment versus TUN only. (**D**) Replicate wells of PCCL3 cells were either untreated or treated with tunicamycin (TUN 0.1 μg/mL) for 24 hours. During the last 12 hours, the cells were also either untreated or treated with a high dose of actinomycin D (5 μg/mL), as indicated. The ER stress–induced rise of *BiP* and *Chop* mRNA levels was almost completely blocked by actinomycin D treatment in the last 12 hours, whereas the increased *Cidea* mRNA level was completely unaffected; error bars represent mean ± SD from 3 independent experiments in which each sample had 3 independent biological replicates; *P < 0.05 versus TUN-treated cells without actinomycin D treatment (1-way ANOVA).

As the effect of ER stress on *Cidea* mRNA (in thyrocytes or other cell types) had not previously been noted to our knowledge, we examined the effect of low-dose actinomycin D (5 ng/mL, for partial inhibition of transcription) on the *Cidea* mRNA response to ER stress in PCCL3 cells. When challenged with a low dose of TUN (0.1 μg/mL), we noted that *BiP* and *Chop* mRNAs that were increased at 12 hours became further increased at 24 hours, and, as expected, responses at both time points were inhibited by partial transcriptional blockade (Figure 5, A and B). In contrast, the level of *Cidea* mRNA by low-dose TUN did not rise between 12 and 24 hours (Figure 5C, red arrow) and was actually augmented by partial transcriptional blockade (Figure 5C). We induced ER stress with cyclopiazonic acid (CPA, a reversible sarco-endoplasmic reticulum Ca^2+^-ATPase pump inhibitor) and then added high-dose actinomycin D (5 μg/mL) to block all transcription, and we observed that whereas the ER-stressed PCCL3 cells maintained *Cidea* mRNA levels between 3 and 12 hours, washout of CPA to reverse ER stress led to a lowering of *Cidea* mRNA (Supplemental Figure 3B). These data suggested that *Cidea* mRNA half-life was shorter in the absence of ER stress and stabilized in the presence of ER stress. If high-dose actinomycin D was added *prior to* the addition of TUN to induce ER stress, then the *Cidea* mRNA elevation was prevented (data not shown). However, when high-dose actinomycin D was added 12 hours *after* initiating an ER stress challenge (with low-dose TUN), induction of *BiP* and *Chop* mRNAs was profoundly suppressed, but the stress-induced elevation of *Cidea* mRNA was not inhibited (Figure 5D). Together, these data suggest that the initial rapid increase of *Cidea* mRNA in response to acute ER stress includes both transcription and mRNA stabilization.

**Figure 6:**
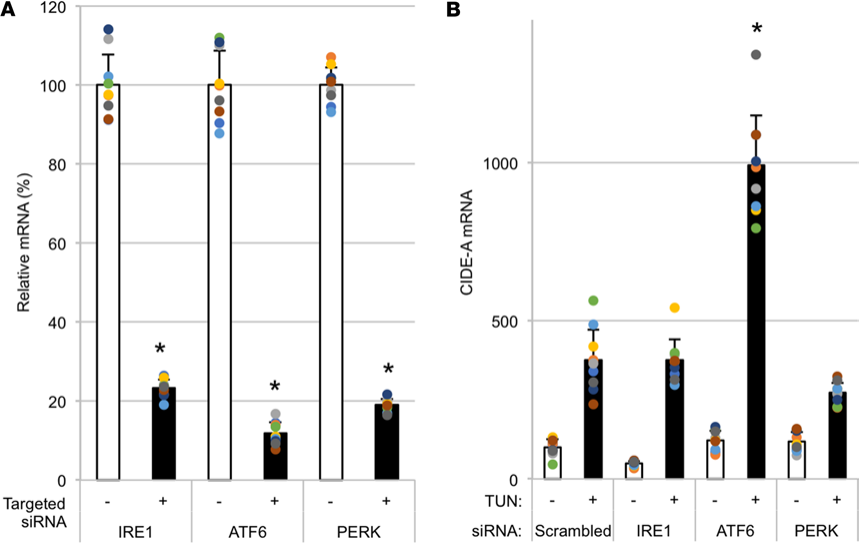
Acute ER stress response after siRNA-mediated knockdown of *Ire1*, *Atf6*, or *Perk* in PCCL3 cells. Replicate wells of PCCL3 were transfected with 30 nM siRNA duplexes for knockdown of *Ire1*, *Atf6*, or *Perk* or scrambled/noncoding oligonucleotides. At 24 hours after oligofection, the cells were treated ± TUN (0.1 μg/mL). After an additional 24 hours, all cells were lysed, and mRNA levels were measured by qRT-PCR for *Ire1*, *Atf6*, and *Perk* (**A**; *P < 0.05 versus scrambled oligo) and *Cidea* (**B**). The data show the mean ± SD (from 3 independent experiments in which each sample had 3 independent biological replicates); *P < 0.05 versus TUN-treated cells transfected with scrambled oligonucleotide (1-way ANOVA).

To examine the relationship of the classical ER stress response to *Cidea* mRNA, we performed individual siRNA-mediated knockdowns of each of the 3 ER stress sensors in PCCL3 cells. Notably, elevation of *Cidea* mRNA in response to acute ER stress (TUN, 24 hours) was not suppressed by knockdown of inositol-requiring enzyme-1 (*Ire1*) or protein kinase R-like ER kinase (*Perk*) and was actually augmented by knockdown of activating transcription factor 6 (*Atf6*) (Figure 6), and these effects could already be detected within 12 hours after addition of TUN to initiate ER stress (Supplemental Figure 4).

### Examination of CIDE-A protein and cell death in ER-stressed thyrocytes.

**Figure 7:**
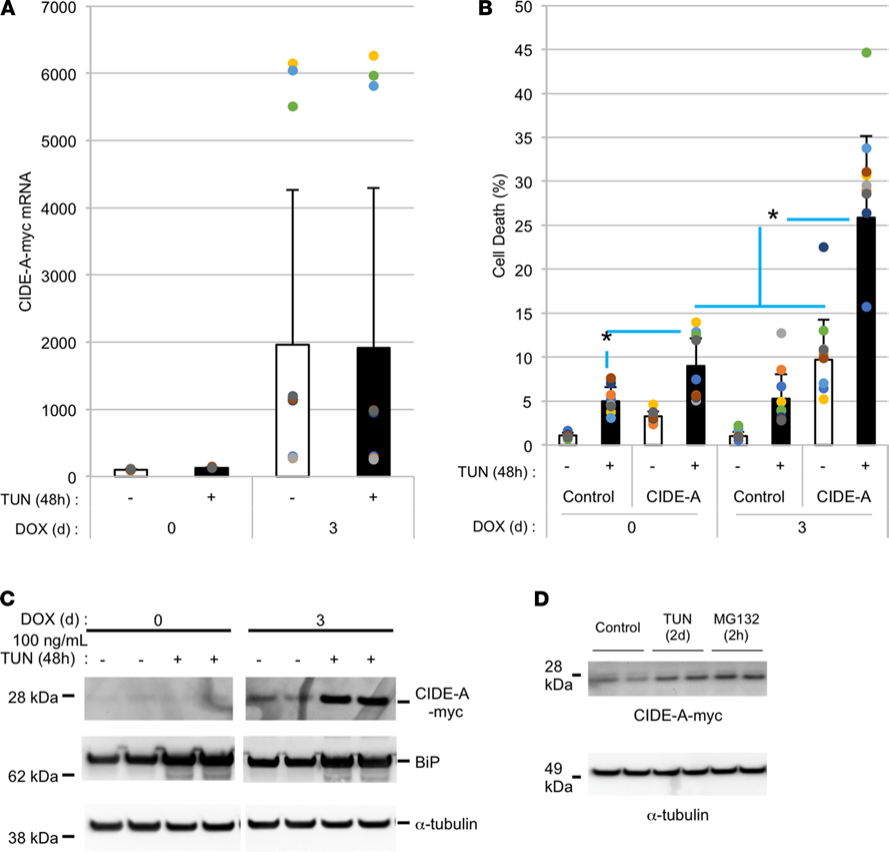
Acute induction of CIDE-A protein in PCCL3 cells, with ER stress, increases cell death. PCCL3 cells with DOX-inducible mouse CIDE-A-myc were prepared as described in Methods. (**A**) In these cells, *Cidea-myc* mRNA was induced with DOX (2 μg/mL, 3 days) and this mRNA was not further increased by ER stress (TUN, 0.1 μg/mL) imposed during the last 48 hours of the 3-day incubation. (**B**) Cell death (by CytoTox-Glo assay) from control PCCL3 cells and those with DOX-inducible CIDE-A, as indicated. In PCCL3 cells with DOX-inducible mouse CIDE-A-myc, TUN alone induced cell death ≤ 5%; DOX alone induced cell death < 8%; TUN + DOX together induced cell death > 20%. *P < 0.05 for the comparator groups shown (1-way ANOVA). (**C**) Immunoblotting of CIDE-A-myc protein levels (upper panel) and BiP levels (middle panel) from the samples in **A**. Tubulin is a loading control (lower panel). (**D**) CIDE-A-myc protein levels in DOX-treated cells for 3 days and treated with TUN (0.1 μg/mL) for the last 2 days or MG132 (10 μg/mL) for the last 2 hours. Tubulin is a loading control (lower panel). The immunoblots of **C** and **D** were repeated 3 times.

**Figure 8:**
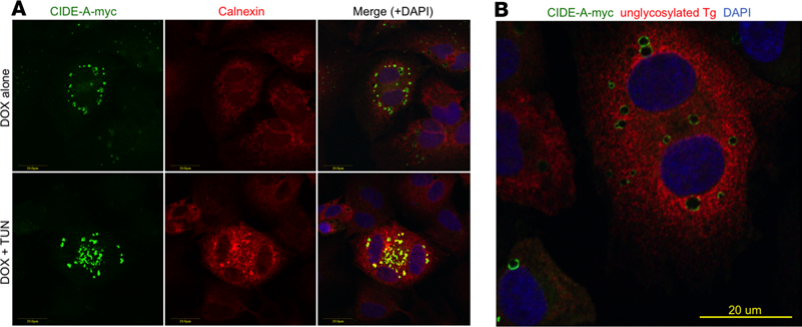
Localization of CIDE-A-myc protein in PCCL3 cells. (**A**) PCCL3 cells transfected to express DOX-inducible mouse CIDE-A-myc were treated with DOX (2 μg/mL) for 3 days ± TUN (for the last 2 days) as in Figure 7. Not all cells in the population expressed CIDE-A-myc. The cells were fixed and immunostained for CIDE-A-myc with anti-myc (shown in green), the ER marker calnexin (shown in red), and nuclei counterstained with DAPI (shown in blue). (**B**) PCCL3 cells treated as in **A**, immunostained for CIDE-A-myc (in green) and unglycosylated misfolded TG that is trapped in the ER (in red) and nuclei counterstained with DAPI (blue). The immunofluorescence experiments were repeated 3 times. Scale bars: 20 μm.

A relationship between CIDE-A and cell death has been repeatedly noted since its initial discovery (8, 10) yet remains poorly understood. We used pTREtight to engineer PCCL3 (rat thyroid) cells to express mouse CIDE-A-myc upon induction by doxycycline (DOX) rather than by ER stress (Figure 7A). In these engineered cells, addition of either TUN (for 2 days) or DOX (for 3 days) induced death in only a small fraction (<10%) of cells, but addition of both treatments induced cell death synergistically (Figure 7B). Interestingly, although ER stress did not increase the recombinant *Cidea-myc* mRNA level, ER stress did increase the recombinant CIDE-A-myc protein level (Figure 7C). By immunofluorescence, the increased CIDE-A-myc protein in PCCL3 thyrocytes exhibited a punctate-like pattern seemingly compatible with that of lipid droplets that emerge from the ER (Figure 8A) and could be distinguished from bulk ER bearing TG that was misfolded upon TUN exposure (Figure 8B). Moreover, CIDE-A-myc protein level was rapidly increased upon addition of the proteasome inhibitor, MG132 (Figure 7D). Thus, the data suggest that ER stress increases and stabilizes *Cidea* mRNA levels and increases CIDE-A protein accompanying increased cell death, which could be limited in cells with siRNA-mediated knockdown of endogenous *Cidea* (Supplemental Figure 5). It has been suggested that lipid droplets may be involved, directly or indirectly, in ER stress and cell death by promoting Ca^2+^ loss from the ER (20). To begin to explore this possibility, PCCL3 cells were transfected to express the ER calcium-sensing fluorescence resonance energy transfer (FRET) probe, D4ER. From this, we observed that TUN-induced ER stress was accompanied by an apparent decrease in ER Ca^2+^ levels, whereas *Cidea* knockdown appeared to protect from the ER stress–mediated loss of ER Ca^2+^ (Supplemental Figure 6).

It has been reported that CIDE-A may be induced as part of a noncanonical proapoptotic stress response in human pancreatic islets (21). In *Akita* diabetic (male) mice, pancreatic islets express misfolded mutant proinsulin (22, 23), and the β cells experience both ER stress and cell death (24). As our laboratory was in possession of reverse-transcribed mRNA samples from isolated pancreatic islets of *Akita* mice at different stages of the disease (based on random blood glucose), we were able to examine islet *Cidea* mRNA levels and noted that these levels rose with worsening hyperglycemia (Supplemental Figure 7).

### Limiting the rise of ER stress–mediated CIDE-A expression correlates with cell survival.

**Figure 9:**
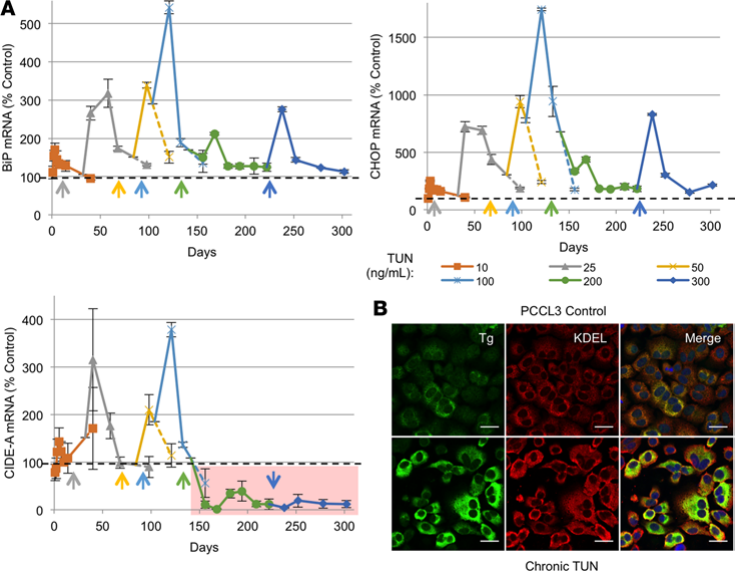
PCCL3 cells that ultimately adapt to chronic TUN exhibit a disproportionately lower relative mRNA level of *Cidea* compared with that of classical ER stress markers. (**A**) PCCL3 cells grown for up to 1 year in stepwise increasing doses of TUN have been described, and the upper 2 graphs show *BiP* and *Chop* mRNA levels in the surviving, growing cells, respectively (for comparison), reproduced from the previous work (reproduced with permission from the *Journal of Biological Chemistry*, ref. 3). The lower graph of **A** shows *Cidea* mRNA levels in the same samples, from the same cells, over the same time course. (**B**) Cells from **A** adapted to a TUN dose of 200 ng/mL were prepared for immunofluorescence with anti-TG (unglycosylated and entrapped in the ER) and anti-KDEL (which reacts with ER-resident proteins, many of which are induced by ER stress response). The immunofluorescence was repeated 3 times.

We recently reported that PCCL3 thyrocytes in progressively increasing doses of TUN nevertheless adapt to ER stress and exhibit long-term survival in culture for up to a year (3). The upper 2 panels of Figure 9A are reproduced with permission from the *Journal of Biological Chemistry* (3); cells adapted to high-dose TUN exhibited enrichment of unglycosylated TG protein entrapped in the ER, along with an increase in the steady-state levels of KDEL-containing ER chaperones and oxidoreductases (Figure 9B). Remarkably, however, upon exposure to the final and highest doses of TUN, the *Cidea* mRNA in these PCCL3 cells was suppressed below control levels and was no longer induced by high-dose TUN challenge (Figure 9A, bottom panel).

**Figure 10:**
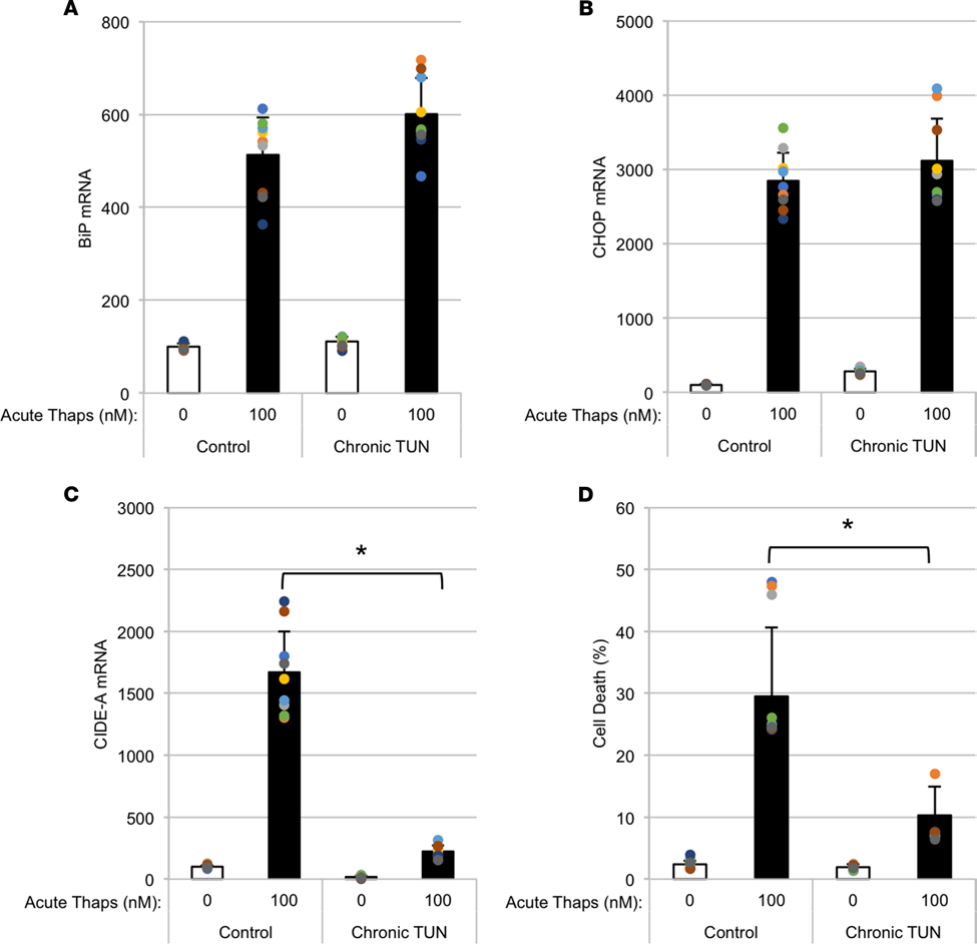
PCCL3 cells that ultimately adapt to chronic TUN exhibit normal acute *BiP* and *Chop* response to Thaps but a blunted *Cidea* response. PCCL3 cells from Figure 9 adapted to grow continuously ± TUN (300 ng/mL) were acutely challenged with Thaps (100 nM) for 48 hours. The cells were then lysed and mRNA levels were measured by qRT-PCR for *BiP* (**A**), *Chop* (**B**), or *Cidea* (**C**). In parallel, cell death was measured at 72 hours by CytoTox-Glo (**D**). The data show the mean ± SD (from 3 independent experiments in which each sample had 3 independent biological replicates for qPCR and 6 for CytoTox-Glo assay); *P < 0.05 for the Thaps response in cells adapted to chronic ER stress versus cells that had not been previously adapted to chronic ER stress (1-way ANOVA).

Having adapted to high-dose TUN, the chronically ER-stressed cells were then exposed an unrelated acute ER stress challenge with Thaps (0.1 μM). Similar to that described above, at 48 hours, control PCCL3 cells induced the mRNAs of *BiP*, *Chop*, and *Cidea* and at 72 hours showed more than 25% cell death (Figure 10, A–D). Remarkably, PCCL3 thyrocytes that had been adapted to chronic ER stress showed no change in their sensitivity of acute response of *BiP* and *Chop* mRNA (Figure 10, A and B) but exhibited a dramatically blunted *Cidea* mRNA response (Figure 10C) accompanied by a significantly blunted cell death response (Figure 10D). Altogether, the data suggest that in addition to its role in the biology of lipid droplets (9), CIDE-A is a novel marker that appears to be linked to a complex, noncanonical, proteotoxic ER stress response program — and limiting its stress-mediated induction has implications for cell survival.

## Discussion

Protein-secreting endocrine tissues exhibit a feature through which when hormone production declines, endocrine circuitry typically activates physiological stimulation of the failing tissue, and in some instances this can lead to a compensatory expansion of endocrine cell mass. In a number of endocrine diseases, including some forms of diabetes mellitus, despite these physiological feedback loops, compensation may fail (25). In normal thyrocytes, ER stress response pathways are already significantly active in cells making physiological levels of wild-type TG (3). Patients (and animals) with congenital hypothyroidism caused by biallelic *TG* mutations resulting in TG protein misfolding trigger further ER stress, yielding both adaptive and cytotoxic stress responses. In such a case, cell death in the thyroid gland may limit the degree of compensatory thyroid gland growth (4). Thyrocytes are a good model for study of ER stress and stress responses because of their exceptional dedication to the synthesis of high levels of TG protein, and the maintenance of thyroid cell mass can be followed both in vivo and in vitro.

ER protein misfolding triggers activation of numerous pathways that can potentially lead to cell death from proteotoxicity (26–28), which underlies the pathogenesis of various neurodegenerative diseases and disorders of the endocrine system (29). A commonly held view is that when cells experience chronic unremitting ER stress, one or more cell-autonomous mechanisms activate cell death (30). Chronic ER stress–mediated cell death has been proposed to be triggered by a variety of alternative mechanisms, including persistently increased CHOP activity (31), ATF4 activity (32), hyperactivated IRE1 (33), JNK activation (34), ER membrane leakage (35), and others. Here, we suggest that additionally, ER stress–mediated cell death might be linked to an inability to suppress the expression of prodeath gene products; conversely, survival might be linked to successful suppression or repression of such genes.

We know that *TG^cog/cog^* thyrocytes experience chronic unremitting ER stress (with a swollen ER accompanied by a markedly activated ER stress response), yet the thyrocytes continue to proliferate (3); however, thyroidal expression of the *TG^rdw^* transgene in *TG^cog/cog^* mice (without changing total *TG* mRNA or the degree of hypothyroidism, and exhibiting comparable levels of canonical ER stress markers) increases the prevailing level of thyrocyte cell death, which correlates with unsuppressed expression of *Cidea* mRNA in thyroid tissue (Figure 2). The effect of acute ER stress to stimulate *Cidea* mRNA levels is not a feature that requires ongoing TSH stimulation of thyrocytes, nor is it a thyrocyte-specific feature. Indeed, *Cidea* transcripts are known to be abundant in white and brown fat as well as mammary tissue; the gene is also expressed at varying levels in heart, lung, skeletal muscle, spleen, thymus, lymphatic tissue, liver, stomach, brain, oocytes, placenta, kidney (36–39), as well as colorectal and prostate cancer cells and endometrial (40) and ovarian cancer cells (41). In our experiments, to exclude any contribution of adipocytes or other cells that reside within the thyroid gland (42), we directly examined the PCCL3 thyrocyte cell line (Figure 3) (or other cell types; Figure 4) to examine the *Cidea* response to ER stress.

*Cidea* is not a classic ER stress response gene (being devoid of an apparent ER stress element or unfolded protein response element in its promoter), and thyrocyte ER stress regulation of *Cidea* exhibits several interesting features. Most notably, separate from the transcriptional regulation that has been reported in other cell types (43), *Cidea* mRNA levels in thyrocytes appear stabilized in the presence of ER stress (Figure 5), while *Atf6* expression under ER stress appears to limit overshoot of *Cidea* mRNA (Figure 6). Clearly, much more needs to be learned about how *Cidea* regulation fits within the broader transcriptomic response to acute proteotoxic ER stress.

Beyond this, in PCCL3 thyrocytes, acute ER stress also elevates intracellular levels of the CIDE-A-myc protein, which otherwise (in unstressed cells) appears to be susceptible to rapid proteasomal degradation (Figure 7, C and D). In addition to acute ER stress enhancing CIDE-A distribution to intracellular organelles suggestive of lipid droplets that emanate from the ER (Figure 8) and influencing ER calcium homeostasis (Supplemental Figure 6), acute ER stress also stimulates cell death (Figure 7B). Conversely, whereas *Cidea* mRNA appears to increase in *Akita* mouse pancreatic islets as the animals progress into worsening diabetes (Supplemental Figure 7), *Cidea* knockdown has been reported to improve survival in ER-stressed pancreatic β cells intoxicated with palmitate (44). CIDE-A–mediated inhibition of cell survival may relate to its downregulation of AMP-kinase activity (45, 46); indeed, increased AMP-kinase activity is thought to be linked to thyrocyte survival in the face of ER stress (3). Remarkably, in contrast with reports of mouse embryonic fibroblasts (47), PCCL3 cells adapted to chronic ER stress unexpectedly did not limit their classical *BiP* or *Chop* response when presented with a new, unrelated, acute ER stress challenge (Figure 10, A and B). Rather, *Cidea* mRNA was no longer elevated above baseline, and *Cidea* exhibited a blunted response to an unrelated acute ER stress challenge (Figure 9A and Figure 10C) accompanied by resistance to ER stress–mediated cell death (Figure 10D).

Importantly, noncanonical mechanisms regulating the transcriptome in response to ER stress have been increasingly recognized, including active suppression of 25%–50% of all ER stress–responsive mRNAs (48). It is highly likely that *Cidea* is regulated as part of a transcriptional and posttranscriptional network of activities, the dissection of which will require further detailed biological and bioinformatics analyses to help account for ER stress–related cell survival and cell death (48).

## Methods

### Materials.

We used mouse anti-CHOP (Santa Cruz Biotechnology sc-7351), anti-eIF2α (Santa Cruz Biotechnology sc-200, sc-11386); rabbit anti-GAPDH (Cell Signaling Technology 2118S), rabbit anti-MYC (Immunology Consultants catalog RMYC-45A-Z), mouse anti-KDEL (Enzo Life Sciences, catalog ADI-SPA-827), mouse anti–α-tubulin (MilliporeSigma, catalog T5168), and rabbit anti-TG and anti-BiP as previously described (13). HRP-conjugated goat anti-rabbit and goat anti-mouse secondary antibodies were from Jackson ImmunoResearch (catalog 111-035-144 and 115-035-174, respectively). TUN and actinomycin D were from MilliporeSigma.

### Generation of TG^cog/cog^ mice bearing transgenic TG^rdw^.

For thyroid-specific expression, a linearized transgene consisting of the bovine *TG* promoter (18) immediately upstream of the full-length mouse *TG* ORF encoding rdw-TG plus a triple-myc tag (i.e., *rdwTg3xMyc*, ref. 15) was injected into B6SJLF2 eggs, obtained by the mating of B6SJLF1 mice (The Jackson Laboratory stock number 100012). Potential transgenic founder animals were screened by PCR of tail snip DNA to generate a 284 bp fragment in the presence of the *TG^rdw^* transgene. Eleven PCR**^+^** founders were repeatedly backcrossed to C57BL/6 and *TG^cog/cog^* mice from The Jackson Laboratory; the 2 strongest expressing lines (by anti-myc Western blotting) were retained. Hypothyroid breeders were given supplemental T_4_ in drinking water provided ad libitum, and supplemental T_4_ was withdrawn at weaning. Animal use was performed in accordance with the University of Michigan’s IACUC.

### TUNEL staining.

TUNEL staining (MilliporeSigma ApopTag In Situ Apoptosis Detection Kit) of mouse thyroid was performed in tissue sections, and nuclei were counterstained with DAPI. Quantitation of images was performed both manually and by using ImageJ (NIH) with the ITCN plugin.

### Thyroid gland size measurement.

Small animal ultrasound (VisualSonics 770) was performed on live anesthetized mice to assess thyroid lobe area (volume data were also obtained but were proportional to area). (Ultrasound-measured areas were ultimately confirmed in euthanized mice by 2-dimensional digital photography of the dissected neck with a calibrated size marker. Both sets of measurements were found to be more reproducible than wet weight of dissected thyroid tissue.)

### Cell death screen.

A mouse apoptosis PCR array (SABiosciences PAMM-012) was used to prescreen 4 *TG^cog/cog^* thyroidal reversed-transcribed RNA samples plus 4 more bearing the *TG^rdw^* transgene, along with positive and negative control genes and plate controls to yield a small data set. Average Ct values were used to calculate the relative mRNA expression of 86 survival or death genes (analysis performed by the University of Michigan Bioinformatics Core; complete data set shown in Supplemental Figure 3A).

### Cell lines.

PCCL3 cells (49) (from B. DiJeso, University of Salento, Lecce, Italy) maintained in DMEM/F12 with 5% fetal bovine serum (FBS), 1 mIU/mL thyrotropin, 1 μg/mL insulin, 5 μg/mL apo-transferrin, 1 nmol/L hydrocortisone (4 hormones obtained from MilliporeSigma), and penicillin/streptomycin. DOX-inducible PCCL3 cells were generated by transfecting pTet-On-Advanced Vector (Clontech) and pTRE-Tight/mCIDE-A-myc into PCCL3 cells (ViaFect, Promega) and selecting for stably transfected clones with 100 μg/mL G418 (Gibco, Thermo Fisher Scientific) and 3 μg/mL puromycin (Clontech), respectively. *Cidea-myc* expression was induced using 2 μg/mL DOX (Clontech). The siRNAs for knockdown of *Ire1*, *Atf6*, and *Perk* in PCCL3 cells were obtained from GE/Dharmacon and transfected using the Lipofectamine RNAiMAX (Thermo Fisher Scientific). Neuro2A (ATCC CCL-131) and NRK cells (ATCC CRL-6509) were grown in DMEM with high glucose (4.5 g/L) plus glutamine and 10% FBS.

### qRT-PCR.

Total RNA was isolated from thyroid tissue or cultured cells using RNeasy Plus (Qiagen), and high-capacity cDNA reverse-transcribed (Thermo Fisher Scientific). Real-time PCR was performed using *Power* SYBR Green Master Mix or TaqMan Fast Advanced Master Mix on a StepOnePlus Real-Time PCR System (Thermo Fisher Scientific). Gene expression was normalized to 18S RNA (mouse tissue) or *B-actin* or *Hprt1* (cells); average Ct values were used to calculate relative mRNA expression. Data are represented as mean ± SD unless otherwise noted, with *P* ≤ 0.05 determined to be significant.

### Western blotting.

Thyroid glands from age-matched mice were disrupted in 10 mM Tris pH 7.4, 150 mM NaCl, 0.1% SDS, 1% NP-40, 2 mM EDTA, and protease inhibitor cocktail (Roche). Cultured cells were lysed in RIPA buffer with Protease and Phosphatase Inhibitor (Pierce Biotechnologies). We loaded comparable levels of total protein from thyroid homogenates or cultured cells (BCA assay, Pierce, Thermo Fisher Scientific), resolved by SDS-PAGE, electrotransferred to nitrocellulose, and immunoblotted, with band detection by SuperSignal West Pico Chemiluminescent Substrate (Pierce Biotechnologies, Thermo Fisher Scientific).

### Immunofluorescence.

Five-micrometer-thick sections of fixed, paraffin-embedded thyroid issues were deparaffinized in Citrisolv (Thermo Fisher Scientific), then rehydrated in a graded series of alcohols, with antigen unmasking in Retrieve-All (Covance) at 90°C for 2 hours. After cooling and washing, sections were blocked for 1 hour with 0.3% Triton X-100, with 10% normal goat serum in PBS. Cultured cells were fixed with 4% formaldehyde, washed twice with PBS, permeabilized with 0.4% Triton X-100 in TBS for 20 minutes (room temperature), and blocked with 3% BSA in 0.2% Triton X-100 in TBS for 1 hour (room temperature). Slides were incubated overnight at 4°C with indicated antibodies. After incubation with secondary antibodies conjugated to Alexa Fluor 488 or Alexa Fluor 555 (Life Sciences, Thermo Fisher Scientific, A-11008 and A-21422, respectively), slides were washed and coverslips mounted with ProLong Gold with DAPI (Life Sciences, Thermo Fisher Scientific), with images captured on an Olympus FV500 confocal microscope.

### Cell death in culture.

Twenty thousand PCCL3 cells were plated in each well (96-well plates) 24 hours before each treatment. Cells were treated as indicated, and both dead cells and total cells were measured by the CytoTox-Glo Cytotoxicity Assay Kit (Promega).

### Statistics.

For analyses of 3 or more groups, 1-way ANOVA with Tukey’s post hoc test was performed, with data shown as mean ± SD. This included *Xbp1* splicing, TUNEL staining, general qPCR (mRNA levels) and *Cidea* mRNA levels, cell death by CytoTox-Glo assay, and FRET ratio. Ultrasound (thyroid area) between the 2 animal groups was analyzed by 2-tailed *t* test. For the screening array plate, the difference between average Ct values of each tested gene was compared with that of control (housekeeping) genes to yield log fold change, and the University of Michigan Bioinformatics Core statistically compared the 2 groups with *P* values adjusted for FDR, with a *P* < 0.05 considered significant.

### Study approval.

Animal experiments were performed under the University of Michigan IACUC–approved animal protocol PRO00008062.

## Author contributions

YM and APK contributed to experimental design, data generation, data analysis, and writing of the manuscript. DL, WC, and SV contributed to data generation and validation and data analysis. LS and ML contributed to supervision and editing the manuscript. PA contributed to experimental design, data analysis, writing of the manuscript, supervision, and funding. All authors discussed results.

## Supplementary Material

Supplemental data

## Version 1 03/04/2021

In-Press Preview

## Version 2 04/08/2021

Electronic publication
