# Supplementary material for: Cell death–associated lipid droplet protein CIDE-A is a noncanonical marker of endoplasmic reticulum stress
Source: JCI Insight. 2021 Apr 8;6(7):e143980. doi: 10.1172/jci.insight.143980 (PMC8119190; doi:10.1172/jci.insight.143980)
Supplement: Supplemental data [file jciinsight-6-143980-s217.pdf]

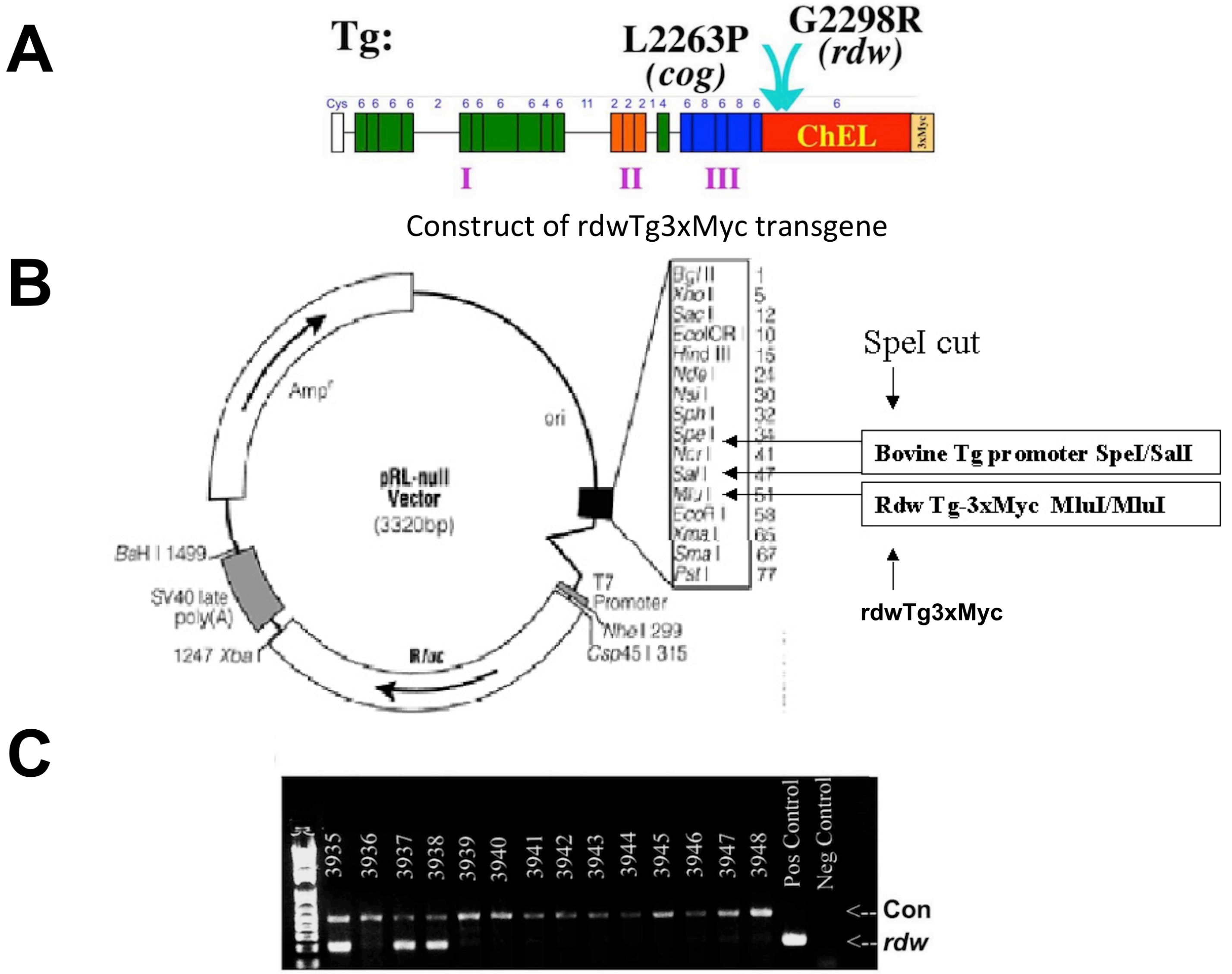

Morishita et al., SI Fig. S1

Figure S1. Generation of *TG*$^{cog/cog}$ mice bearing a *TG*^{rdw} transgene. A. Depiction of the Tg primary structure, indicating regions I, II, III, ChEL domain, and the location of a triple-myc epitope tag at the C-terminus. The positions of *cog* and *rdw* mutations within the Tg-ChEL domain are indicated. **B.** Construction of a plasmid bearing the bovine *TG* promoter followed by the ORF encoding rdw-Tg-3xMyc. **C.** PCR identification of the *TG*^{rdw} transgene specified by a 284 bp PCR fragment amplified from genomic DNA; a 500 bp beta-actin amplicon was included as an internal control (Con) for the PCR reaction.

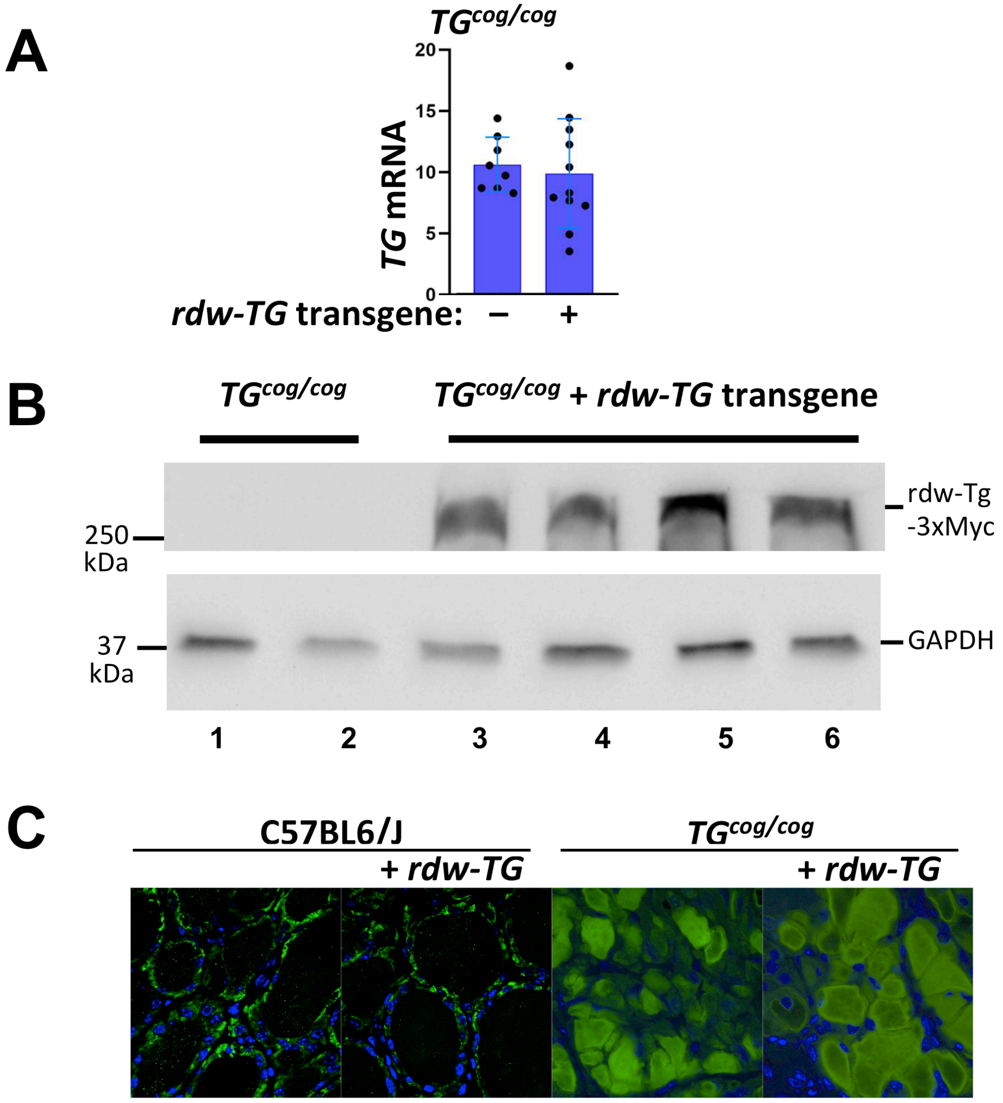

Morishita et al., SI Fig. S2

Figure S2. Characterization of the thyroid gland of $TG^{cog/cog}$ mice bearing the TG^{rdw} transgene. A. Total *TG* mRNA in the thyroid gland of $TG^{cog/cog}$ mice lacking or bearing the TG^{rdw} transgene, normalized to housekeeping RNA ± s.d.. **B.** Western blot detection of rdw-Tg-3xMyc in $TG^{cog/cog}$ mice bearing the TG^{rdw} transgene, and nontransgenic controls. GAPDH is a loading control. **C.** BiP immunofluorescence in the thyroid glands of 2 month-old control mice lacking or bearing the TG^{rdw} transgene, or $TG^{cog/cog}$ mice lacking or bearing the TG^{rdw} transgene (magnification identical in all four panels; but the ER is vastly swollen in $TG^{cog/cog}$ mice).

A

Symbol	Gene Name	Log F.C.	Fold Decrease	Fold Increase	p-value
Casp14	Caspase 14	1.7193956		3.293	0.63272087
Pak7	P21 (CDKN1A)-activated kinase 7	1.6116381		3.056	0.685051579
Casp4	Caspase 4, apoptosis-related cysteine peptidase	0.94105285		1.92	0.363611445
Naip2	NLR family, apoptosis inhibitory protein 2	0.7849496		1.723	0.34835015
Bcl2l10	Bcl2-like 10	0.7774892		1.714	0.8294774
Cd40	CD40 antigen	0.67228735		1.594	0.398012038
Tnf	Tumor necrosis factor	0.6281711		1.545	0.34835015
Fasl	Fas ligand (TNF superfamily, member 6)	0.60931435		1.526	0.63272087
Casp1	Caspase 1	0.4811991		1.396	0.543635
Casp12	Caspase 12	0.3057911		1.236	0.63272087
Birc3	Baculoviral IAP repeat-containing 3	0.29844685		1.23	0.407350449
Birc5	Baculoviral IAP repeat-containing 5	0.2969796		1.229	0.63272087
Traf3	Tnf receptor-associated factor 3	0.24440835		1.185	0.602356862
Il10	Interleukin 10	0.19356275		1.144	0.87947525
Fas	Fas (TNF receptor superfamily member 6)	0.12996085		1.094	0.752991837
Trp73	Transformation related protein 73	0.121524133		1.088	0.911164404
Dad1	Defender against cell death 1	0.09687685		1.069	0.911164404
Tsc22d3	TSC22 domain family, member 3	0.07626085		1.054	0.888121482
Hells	Helicase, lymphoid specific	0.05592685		1.04	0.877025045
Cd70	CD70 antigen	0.04265235		1.03	0.967392141
Traf1	Tnf receptor-associated factor 1	0.0385156		1.027	0.911164404
Ripk1	Receptor (TNFRSF)-interacting serine-threonine kinase 1	-0.00652765	1.004534874		0.975777964
Casp2	Caspase 2	-0.0522054	1.036848709		0.877025045
Casp6	Caspase 6	-0.1038694	1.074651886		0.752991837
Bok	BCL2-related ovarian killer protein	-0.11176065	1.080546119		0.877025045
Bag1	Bcl2-associated athanogene 1	-0.11594015	1.08368101		0.706842589
Casp7	Caspase 7	-0.1186809	1.085741682		0.830261966
Cd40lg	CD40 ligand	-0.1200289	1.086756632		0.911164404
Nfkb1	Nuclear factor of kappa light polypeptide gene enhancer in B-cells 1, p10	-0.1287844	1.093372049		0.685051579
Tnfsf10	Tumor necrosis factor (ligand) superfamily, member 10	-0.1362834	1.099070095		0.8294774
Api5	Apoptosis inhibitor 5	-0.1437264	1.104754954		0.685051579
Mcl1	Myeloid cell leukemia sequence 1	-0.1462704	1.10670476		0.706842589
Bcl2	B-cell leukemia/lymphoma 2	-0.14710865	1.107347976		0.685051579
Cradd	CASP2 and RIPK1 domain containing adaptor with death domain	-0.14720365	1.107420896		0.752991837
Trp53	Transformation related protein 53	-0.15323265	1.112058474		0.63272087
Tnfsf12	Tumor necrosis factor (ligand) superfamily, member 12	-0.15686215	1.114859688		0.63272087
Cideb	Cell death-inducing DNA fragmentation factor, alpha subunit-like effecto	-0.17342015	1.127728788		0.712831822
Prdx2	Peroxiredoxin 2	-0.17391265	1.128113832		0.602356862
Bak1	BCL2-antagonist/killer 1	-0.1783204	1.131565738		0.624430267
Pim2	Proviral integration site 2	-0.19097665	1.141536231		0.690660276
Nme5	Non-metastatic cells 5, protein expressed in (nucleoside-diphosphate kin	-0.1964899	1.145906951		0.87947525
Bnip2	BCL2/adenovirus E1B interacting protein 2	-0.2119389	1.158243752		0.593058608
Traf2	Tnf receptor-associated factor 2	-0.2144489	1.160260618		0.71396469
Ltbr	Lymphotoxin B receptor	-0.22269997	1.166915396		0.588281356
Xiap	X-linked inhibitor of apoptosis	-0.22709815	1.170478269		0.63272087
Polb	Polymerase (DNA directed), beta	-0.22793965	1.171161189		0.588281356
Bnip3	BCL2/adenovirus E1B interacting protein 3	-0.22835215	1.171496099		0.690660276
Bad	BCL2-associated agonist of cell death	-0.2283589	1.17150158		0.63272087
Tnfrsf1a	Tumor necrosis factor receptor superfamily, member 1a	-0.2319194	1.174396359		0.602356862
Bid	BH3 interacting domain death agonist	-0.2410539	1.181855701		0.488991417
Tnfrsf11b	Tumor necrosis factor receptor superfamily, member 11b (osteoprotege	-0.2424994	1.183040448		0.63272087
Apaf1	Apoptotic peptidase activating factor 1	-0.25016865	1.189346141		0.588281356
Bax	Bcl2-associated X protein	-0.26241415	1.199484197		0.588281356
Trp63	Transformation related protein 63	-0.2777569	1.212308523		0.63272087
Nod1	Nucleotide-binding oligomerization domain containing 1	-0.2861969	1.21942151		0.588281356
Rnf7	Ring finger protein 7	-0.28647315	1.21965503		0.407350449
Birc2	Baculoviral IAP repeat-containing 2	-0.3003984	1.231484441		0.588281356
Cflar	CASP8 and FADD-like apoptosis regulator	-0.3117139	1.241181329		0.476792335
Dffa	DNA fragmentation factor, alpha subunit	-0.3122519	1.241644268		0.496656571
Casp3	Caspase 3	-0.3712414	1.293465344		0.407350449
Lhx4	LIM homeobox protein 4	-0.3834164	1.304427173		0.690660276
Bcl2l2	Bcl2-like 2	-0.38982565	1.310235052		0.63272087
Casp8	Caspase 8	-0.39253115	1.312694454		0.407350449
Dffb	DNA fragmentation factor, beta subunit	-0.3927669	1.312908978		0.363611445
Sphk2	Sphingosine kinase 2	-0.40093315	1.320361658		0.476792335
Akt1	Thymoma viral proto-oncogene 1	-0.4028944	1.322157824		0.407350449
Bcl10	B-cell leukemia/lymphoma 10	-0.4079989	1.326844129		0.476792335
Naip1	NLR family, apoptosis inhibitory protein 1	-0.42441893	1.342031871		0.8294774
Nol3	Nucleolar protein 3 (apoptosis repressor with CARD domain)	-0.4294259	1.346697571		0.34835015
Trp53inp1	Transformation related protein 53 inducible nuclear protein 1	-0.4321424	1.349235703		0.398012038
Trp53bp2	Transformation related protein 53 binding protein 2	-0.45523115	1.371002451		0.363611445
Atf5	Activating transcription factor 5	-0.46433315	1.379679483		0.588281356
Bnip3l	BCL2/adenovirus E1B interacting protein 3-like	-0.49651265	1.410799187		0.34835015
Casp9	Caspase 9	-0.50054865	1.414751483		0.34835015
Bcl2l1	Bcl2-like 1	-0.5441219	1.458132578		0.34835015
Bag3	Bcl2-associated athanogene 3	-0.5545094	1.468669116		0.34835015
Card6	Caspase recruitment domain family, member 6	-0.56198015	1.476294093		0.34835015
Tnfrsf10b	Tumor necrosis factor receptor superfamily, member 10b	-0.5689564	1.483450101		0.34835015
Fadd	Fas (TNFRSF6)-associated via death domain	-0.6775089	1.599375729		0.34835015
Zc3hc1	Zinc finger, C3HC type 1	-0.7236599	1.651365992		0.34835015
Dapk1	Death associated protein kinase 1	-0.7529434	1.685227543		0.398012038
Card10	Caspase recruitment domain family, member 10	-0.7724839	1.70820829		0.363611445
Pycard	PYD and CARD domain containing	-0.9724674	1.962193611		0.421813196
Cidea	Cell death-inducing DNA fragmentation factor, alpha subunit-like effecto	-2.4799494	5.578778996		0.407350449

B

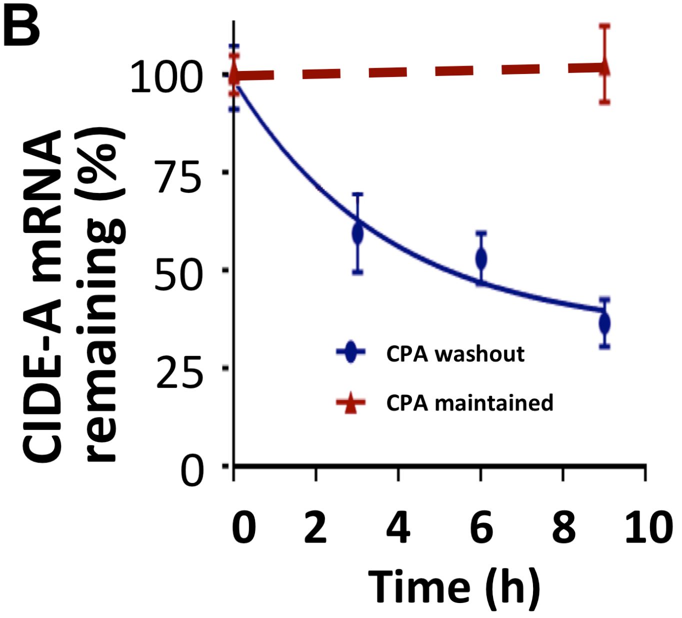

Morishita et al., SI Fig. S3

Figure S3. Characterization of the thyroid gland of *TG*$^{cog/cog}$ mice bearing the *TG*^{rdw} transgene. A. Reversed-transcribed RNA from the thyroid glands of four *TG*$^{cog/cog}$ mice were compared to four bearing the *TG*^{rdw} transgene on Cell Death Array plates (SA Biosciences, #PAMM-012). Average CT values were used to calculate the relative mRNA expression of the 86 survival or death genes. The most upregulated mRNA in transgenic animals [shown as downregulated (in red) in nontransgenic *TG*$^{cog/cog}$ animals] was CIDE-A. **B.** ER stress was induced in PCCL3 cells by treatment with CPA (25 µM) for 3 h. At that time, high-dose actinomycin D (5 µg/mL) was added and CPA was either continued for an additional 3 h (red line) or the cells washed and media changed in the absence of CPA (blue line) — time points are shown. At each time point, the cells were lysed, and CIDE-A mRNA level quantified (relative to HPRT1). The data represent the mean (from three independent biological replicates) ± s.d. within a single experiment.

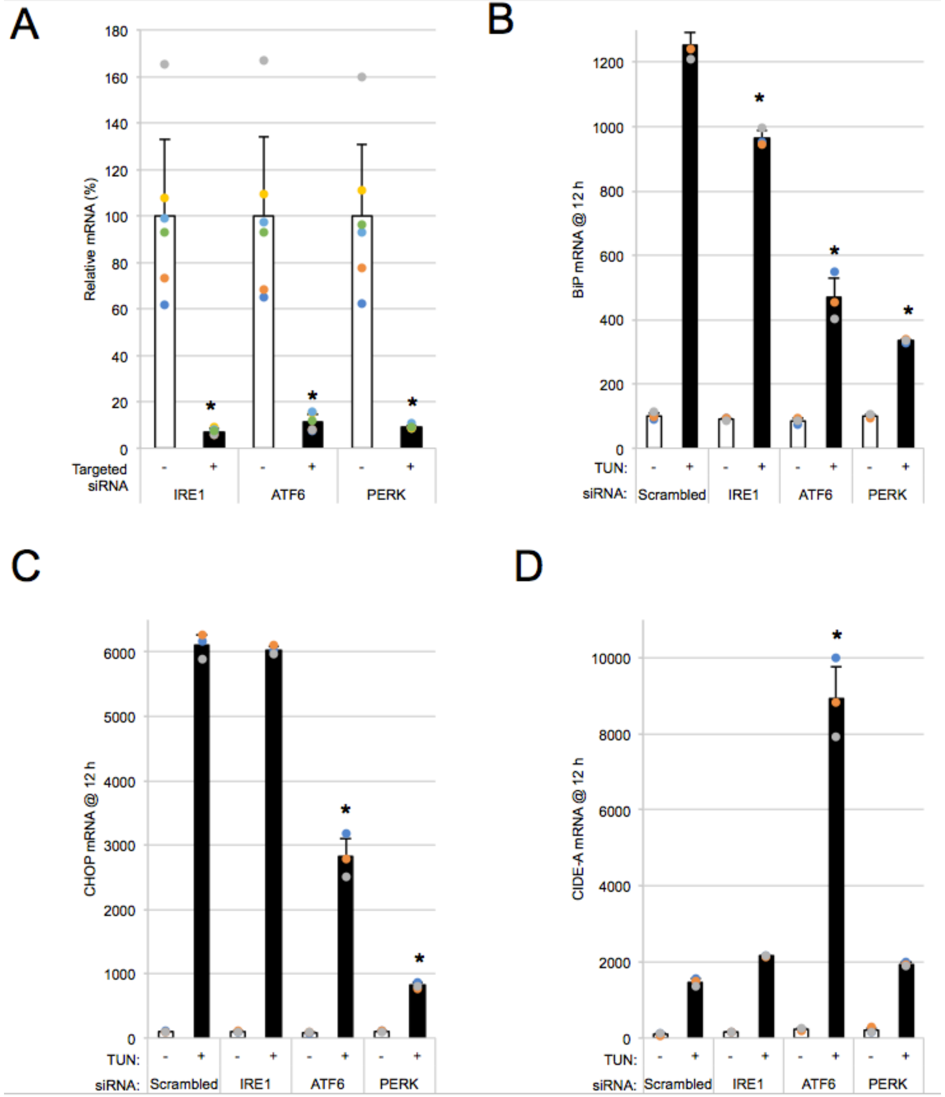

Figure S4. Acute ER stress response after siRNA-mediated knockdown of IRE1, ATF6, or PERK in PCCL3 cells. Replicate wells of PCCL3 were transfected with 30 nM siRNA duplexes for knockdown of IRE1, ATF6, or PERK, or scrambled/noncoding oligos. At 24 h after oligofection, the cells were treated ± TUN (0.1 µg/mL). After an additional 12 h, all cells were lysed, and mRNA levels were measured by q-rtPCR for IRE1, ATF6 and PERK (**panel A**; * $p < 0.05$ versus scrambled oligo), BiP (**panel B**), CHOP (**panel C**), and CIDE-A (**panel D**). The data show the mean (from three biological replicates) ± s.d in a single experiment; * $p < 0.05$ versus TUN-treated cells transfected with scrambled oligo (*decreased* for BiP and CHOP; *increased* for CIDE-A).

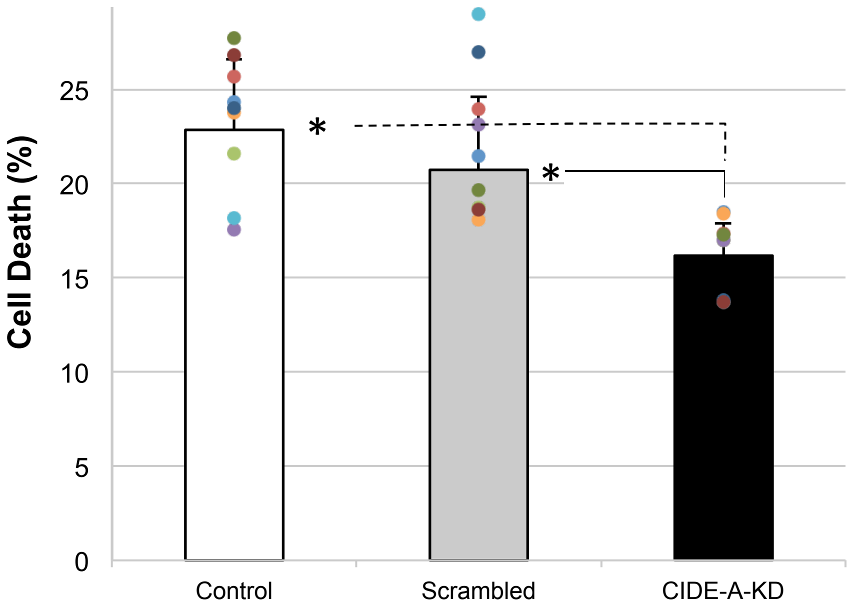

Figure S5. Cell death limited by siRNA-mediated knockdown of CIDE-A. Each set of cells were either untransfected (Control) or transfected with Scrambled or siRNA-mediated knockdown of endogenous CIDE-A (CIDE-A-KD). All cells were treated with DOX (for the full 3 d) to induce CIDE-A-myc (as in Fig. 7) plus TUN (0.1 μg/mL for the last 2 d) to induces ER stress. At 3 d, cell death was quantified in all samples; cell death was limited by CIDE-A-KD; the data represent the mean (from three independent experiment in which each experimental sample had three biological replicates) ± s.d.; * $p < 0.05$ versus samples without CIDE-A-KD.

Morishita et al., Figure. S5

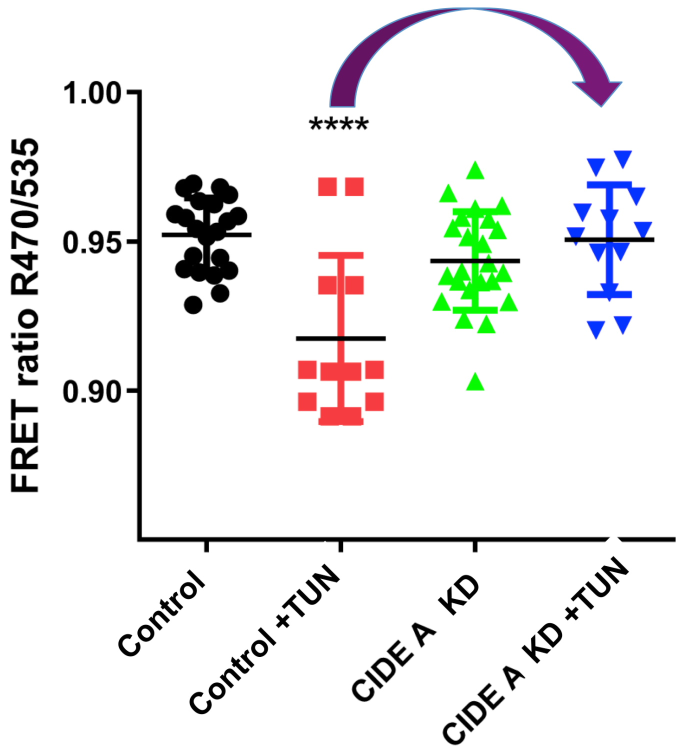

Morishita et al., SI Fig. S6

Figure S6. ER stress effect on ER calcium level in PCCL3 thyrocytes. One day after plating, PCCL3 cells were transfected with Lipo-RNAiMax bearing either 10 nM scrambled oligo (*left-two set of samples*) or siRNA for knockdown of CIDE-A (*right-two set of samples*). Thereafter, all sets of cells were plasmid-transfected (Viafect transfection reagent) to express D4ER in pCDNA3.1. One half of each set of cells were challenged with TUN (0.1 µg/mL), or vehicle alone. At 48 h after TUN addition, transfected cells were washed in PBS plus 2 mM $MgCl_2$ and 5.5 mM glucose, and underwent fluorescence imaging and FRET analysis using 430 nm excitation and 470/535 nm ratiometric emission. The FRET ratio shown for each cell (acquired using Metafluor) reflects the relative Ca^{++} level in the ER; a lower ratio signifies lower ER calcium. Each point reflects a single cell from a representative experiment (of four independent FRET experiments); mean values ± s.d. are shown.

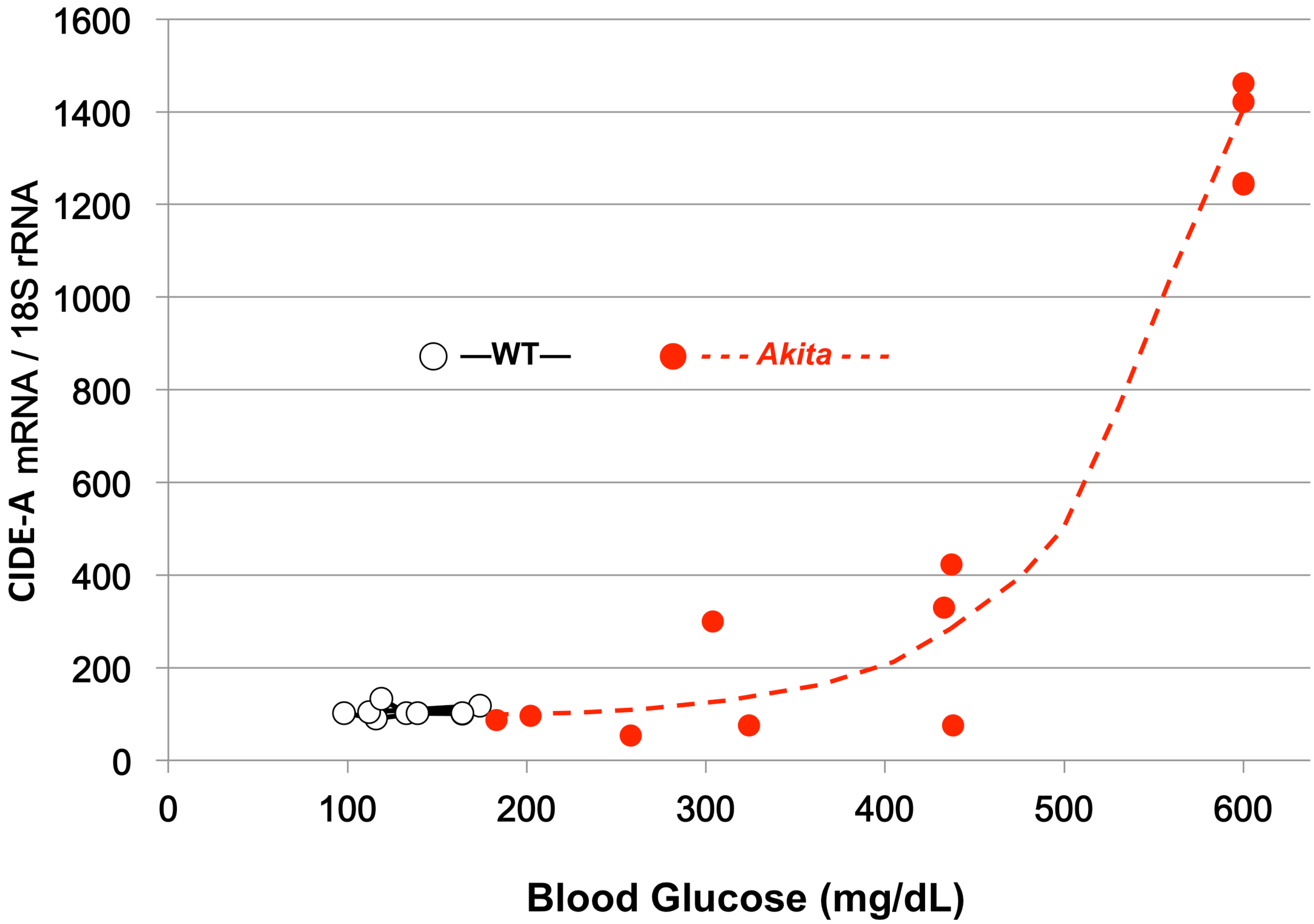

Figure S7. CIDE-A mRNA levels in *Akita* mouse pancreatic islets as a function of progressive dysglycemia. Islets were isolated from male wild-type and *Akita* mice that reflect progression based on random blood glucose. Each point represents a different animal, with CIDE-A mRNA levels normalized to ribosomal 18S RNA.
